# Genetic Polymorphisms of Peroxisome Proliferator-Activated Receptors and the Risk of Cardiovascular Morbidity and Mortality in a Community-Based Cohort in Washington County, Maryland

**DOI:** 10.1155/2008/276581

**Published:** 2007-12-04

**Authors:** L. Gallicchio, Bindu Kalesan, Han-Yao Huang, Paul Strickland, Sandra C. Hoffman, Kathy J. Helzlsouer

**Affiliations:** ^1^Prevention and Research Center, Weinberg Center for Women's Health and Medicine, Mercy Medical Center, 227 Street Paul Place, 6th Floor, Baltimore, MD 21202, USA; ^2^Department of Epidemiology, Johns Hopkins Bloomberg School of Public Health, 615 North Wolfe Street, Baltimore, MD 21205, USA; ^3^Department of Environmental Health Sciences, Johns Hopkins Bloomberg School of Public Health, 615 North Wolfe Street, Room E7535, Baltimore, MD 21205, USA

## Abstract

The primary aim of this study was to examine prospectively the associations between 5 peroxisome proliferator-activated receptor (*PPAR*) single nucleotide polymorphisms (SNPs) and cardiovascular morbidity and mortality in a community-based cohort study in Washington County, Maryland. Data were analyzed from 9,364 Caucasian men and women participating in CLUE-II. Genotyping on 5 *PPAR* polymorphisms was conducted using peripheral DNA samples collected in 1989. The followup period was from 1989 to 2003. The results showed that there were no statistically significant associations between the *PPAR* SNPs and cardiovascular deaths or events. In contrast, statistically significant age-adjusted associations were observed for PPARG rs4684847 with both baseline body mass and blood pressure, and for PPARG rs709158, PPARG rs1175543, and PPARD rs2016520 with baseline cholesterol levels. Future studies should be conducted to confirm these findings and to explore the associations in populations with greater racial and ethnic diversity.

## 1. INTRODUCTION

The peroxisome proliferator-activated receptors (*PPAR*s) are part of a superfamily of ligand-activated transcription factors involved in fatty acid oxidation and lipid metabolism [1]. Three distinct isoforms of *PPAR*s that are encoded by separate genes have been identified: *PPAR*-*α*, *PPAR*-*γ*, and *PPAR*-*δ* [2]. The three isoforms play distinct physiological roles depending on their tissue distribution. *PPAR*-*α*, which is expressed in the liver, heart, skeletal muscle, and kidney, regulates lipid and lipoprotein metabolism. *PPAR*-*γ* is expressed in white and brown adipose tissue and is involved in adipocyte differentiation, lipid storage, and glucose metabolism. *PPAR*-*δ* is expressed in many tissues and stimulates fatty acid oxidation [2, 3]. Beyond these major roles, *PPAR*s also have been shown to play a role in other biological processes, including the regulation of inflammatory and oxidative pathways [2].

*PPAR*s are found in endothelial and vascular smooth muscle cells and have been shown to influence inflammatory, fibrotic, and hypertrophic responses in the heart and vascular wall [4]. Because of their location and their involvement in fatty acid oxidation, lipid metabolism, and inflammation, the role of *PPAR*s in cardiovascular disease and risk factors of cardiovascular disease has been of great interest. In general, activation of the *PPAR*s, both naturally and synthetically, is considered beneficial for cardiovascular health [2]. Both PPAR-*α* and PPAR-*γ* play a role in modulating atherosclerosis; for example, PPAR-*γ* activation may promote monocyte apoptosis, contributing to the stabilization of atherosclerotic lesions [5, 6]. Further, clinical trials have shown that the use of pharmacological *PPAR* agonists such as fibrates (*PPAR*-*α* agonist) is antiatherogenic. Fibrates elevate high-density lipoprotein (HDL) levels, decrease low-density lipoprotein (LDL) and triglyceride levels, and reduce an individual’s risk of experiencing a cardiac event [7].

A number of *PPAR* polymorphisms have been identified within the 3 *PPAR* isoforms and there is a considerable amount of literature on the associations between these polymorphisms and cardiovascular risk factors (reviewed in Cresci [7]). There are less data, however, on the associations between the *PPAR* polymorphisms and cardiovascular disease events (e.g., myocardial infarction (MI), cardiovascular-related death). Further, findings with regard to these associations have been inconsistent. For example, a case-control study nested within the Physician’s Health Study suggested that the *PPARG* Pro12Ala polymorphism, located in exon B of *PPAR*-*γ*, is associated with a reduced risk of MI [8]. In contrast, a recent publication using data from the Health Professionals Followup Study showed that male carriers of the Ala12 allele had an increased risk of MI or cardiac death [9] while other studies have observed no statistically significant association between *PPARG* Pro12Ala and cardiovascular events or death [9, 10]. Thus, additional studies of these polymorphisms are necessary to help us better understand the role of *PPAR* genetics in cardiovascular disease especially in light of available pharmacological *PPAR* targeted agents.

The primary aim of this study was to examine prospectively the associations between 5 *PPAR* polymorphisms (4 in *PPAR*-*γ*: rs4684847, rs709158, rs1175543, and rs1801282; and 1 in *PPAR*-*δ*: rs2016520) and cardiovascular morbidity and mortality in a community-based cohort study in Washington County, Maryland. As a secondary aim, we also examined the associations between the *PPAR* polymorphisms and cardiovascular risk factors.

## 2. METHODS

### 2.1. Study sample

In 1974 and 1989, two cohorts named CLUE I and CLUE II (“*Give us a Clue to Cancer and Heart Disease*”) were established in Washington County, Maryland. CLUE I and CLUE II enrolled 20 305 and 25 081 Washington County residents, respectively. At baseline for both cohorts, participants provided informed consent, completed a brief questionnaire, and donated a blood sample. The questionnaire ascertained data on age, gender, marital status, education, height and weight (CLUE II only), cigarette smoking, and medication and vitamin supplement use within the 48 hours prior to blood donation. In addition, in both 1974 and 1989, blood pressure was measured by a study nurse with a blood pressure cuff while the participant was in a seated position. Blood pressure was assessed three times in succession and the third blood pressure value was recorded. In 1989, total cholesterol (nonfasting) was assayed. Individuals who donated blood to both CLUE I and CLUE II constitute the Odyssey cohort (*N* = 8394) [11, 12].

In addition to the Odyssey cohort, a CLUE II subcohort was selected for case-cohort studies that would be conducted using the CLUE II cohort data. The subcohort was identified by taking an approximate 10% age- and sex-stratified random sample of CLUE II participants who donated a blood specimen and were adult residents of Washington County, Maryland. Of the 2460 participants identified for the subcohort, 807 were also in the Odyssey cohort. Therefore, 10 047 unique participants were part of either the Odyssey cohort or the randomly selected CLUE II subcohort.

Of the participants in the Odyssey Cohort and the CLUE II subcohort, DNA was successfully extracted from the buffy coat samples of 9960 individuals (99.1%). DNA from these participants was genotyped for polymorphisms in genes controlling biological processes such as inflammation that have been associated with multiple diseases. For the study presented here, 5% of the Odyssey and subcohort participants who had no data on all of the chosen *PPAR* SNPs (*n* = 475) were excluded from the analysis. Further, all non-Caucasians (*n* = 121) were excluded from the analysis because previous studies have shown that race is an important effect modifier in investigations of polymorphisms and disease and there were not a sufficient number of non-Caucasians in the cohort to analyze the associations among this group. With the exception of race, excluded and included participants did not differ with respect to baseline characteristics. This study was approved by the Institutional Review Board of the Johns Hopkins Bloomberg School of Public Health.

### 2.2. Outcome assessment

**Mortality**

All participants were followed from the date of blood draw to the date of death or the end of follow up (August 31, 2003), whichever came first. In the CLUE cohorts, deaths are identified through daily searches of obituaries, cross-linkage with death certificates for Washington County, and through searches of the Social Security Administration for individuals aged 65 or older and the National Death Index. Cause of death is ascertained from the underlying cause on Maryland State death certificates as coded by state nosologists. Of specific interest in this study were cardiovascular disease deaths, for which the underlying cause was coded as ICD-9 390–459 or ICD-10 I00–I99. During the followup period, 2159 deaths were documented in the Odyssey cohort and the CLUE II subcohort, and of these, 791 (36.6%) were cardiovascular deaths. Approximately 4% (*n* = 334) of the Odyssey cohort and the CLUE II subcohort participants were lost to follow up. Since these individuals were not documented to have died during the followup period, they were considered alive at the end of follow up and censored at August 31, 2003.

**Morbidity**

Information on cardiovascular events was obtained using participant self-report beginning with questionnaires administered in 1996 and about every 2 years thereafter. On these questionnaires, participants were asked whether a “doctor had told them they ever had” a specific condition and at what age the condition was first diagnosed. The cardiovascular-related outcomes queried were: diabetes, high blood pressure, high cholesterol, heart attack (MI), angina pectoris, stroke, transient ischemic attack, peripheral artery disease, arrhythmia, and blood clots. Data were examined across the questionnaires for consistency; 5% of the participants had inconsistent data with regards to self-reported events. However, exclusion of these participants did not change the results and, therefore, these participants were not excluded. For this analysis, we examined any self-reported nonfatal cardiovascular event as an outcome. A nonfatal cardiovascular event was defined as consistent reporting from 1996 to 2003 of any one of the following cardiovascular conditions: MI, angina, stroke, transient ischemic attack, peripheral arterial disease, arrhythmia, or blood clots. We also considered a composite variable including only MI, stroke, transient ischemic attack, and peripheral arterial disease; however, the results were similar to the composite variable including all seven outcomes and, therefore, the seven outcome variable was used in all analyses. Individual diagnoses were also examined separately. Because of the inconsistency in the collection of data on the age at which a condition occurred as well as the large amount of missing data for the age variables, age at diagnosis data were not used in the analysis.

### 2.3. Genotyping

The *PPAR* SNPs analyzed in this study were a part of a larger group of 210 SNPs selected for investigation within the Odyssey cohort. SNPs were selected based on the following criteria: (a) the minor allele frequency was estimated to be ≥5% among Caucasians in the published literature or databases; (b) the polymorphism was in a gene of known or of promising importance in the development of cancer, cardiovascular diseases, and/or longevity; and (c) the polymorphism was either known to be functional or was likely to alter function based on the published literature. *PPAR* polymorphisms selected for analysis were rs2016520 (*PPARD* Ex4 + 15C > T), rs709158 (*PPARG* IVS9 + 4523A > G), rs1175543 (*PPARG* IVS9 + 7780A >G), rs1801282 (*PPARG* Pro12Ala), and rs4684847 (*PPARG* IVS3-6622C > T). To note, none of the other 210 SNPs selected for investigation in the Odyssey cohort were located in PPAR-*α*, PPAR-*γ*, or PPAR-*δ*.

DNA extracted from the preserved buffy coat samples collected in 1989 were used for genotyping. Within 6 hours of collection, the heparinized blood sample was centrifuged at 1500g for 30 minutes at room temperature. Blood samples were separated into plasma, buffy coat, and red blood cells and frozen at −70ºC within 24 hours of collection. The buffy coat remained frozen until DNA extraction was performed. The DNA extraction procedures used the alkaline lysis method [13]. Genotyping was performed by Celera Genomics Co. (Rockville, Md, USA) for rs4684847, rs709158, and rs1175543 and by Applied Biosystems Inc. (Foster City, Calif, USA) for rs2016520 and rs1801282. All polymorphisms were genotyped using TaqMan technology. Laboratory technicians were masked to disease status. Of the 9,364 participants in the analytic cohort, approximately 90% had data on all five genotypes; 6.7% had data on four, 2.4% had data on three, 0.7% had data on two, and 0.07% had data on only one.

### 2.4. Statistical analysis

The Hardy-Weinberg equilibrium for each SNP was tested by a goodness-of-fit approach. As reported in separate publication, all of the *PPAR* SNPs were in Hardy-Weinberg equilibrium [12]. The cohort characteristics were stratified by gender and compared using chi-square tests or student t-tests. Blood pressure at baseline was categorized into 3 groups independent of antihypertensive medication use as follows: normal, individuals with a systolic pressure less than 120 and diastolic pressure less than 80; hypertensive, those with systolic pressure greater than 140 or diastolic pressure greater than 90; and prehypertensive, those with a systolic pressure between 120 and 140 or diastolic pressure between 80 and 90. The age-adjusted associations between the *PPAR* SNPs and cardiovascular risk factors (i.e., baseline BMI, cholesterol levels, blood pressure) were examined using logistic regression models. Age was adjusted for in all analyses as there were statistically significant age differences for several of the SNPs. Gender was not adjusted for in these analyses because it was not associated with SNP prevalence. Cox-proportional hazard ratios were calculated for both all-cause and cardiovascular mortality after adjustment for age. Since the nonfatal cardiovascular outcomes (including MI), followed a Poisson distribution, and age at diagnosis data were not used in the analysis, age-adjusted relative risks for nonfatal cardiovascular events and for only MI were obtained using Poisson regression methods; this type of analysis was also used when analyzing fatal and nonfatal outcomes combined. Premature death (both overall and due to cardiovascular disease) was also examined as an outcome variable and defined as death prior to the age of 65. All analyses were done separately for the 5 SNPs and stratified by gender, diabetes diagnosis, and body mass index (BMI) at baseline. No differences in the risk estimates were observed in these strata and, therefore, only results for the entire cohort are presented.

To address the issue of multiple testing in this study, *P* values for the associations between SNPs and the cardiovascular risk factors were adjusted for the false discovery rate utilizing Fisher’s combination method using bootstrap resampling. All statistical analysis was carried out using SAS software, version 9.1 (SAS Institute, Inc., Cary, NC, USA). A two-sided *P* value ≤.05 was considered statistically significant.

## 3. RESULTS

Baseline characteristics of the study sample, overall and by gender, are shown on Table 1. In 1989, males were significantly more likely than females to have some college education, to report being a current or former smoker, and to be categorized as prehypertensive or hypertensive. In addition, males had a significantly higher mean BMI than females. In contrast, females were more likely to have cholesterol levels greater than 200 mg/dL, either with or without cholesterol medication use, than males. There were no gender differences in baseline age or *PPAR* SNP prevalence.

Table 2 shows the age-adjusted associations between the *PPAR* SNPs and cardiovascular risk factors examined at baseline and at follow up. After adjustment for age, BMI at baseline was significantly associated with the *PPARG* rs4684847 SNP such that individuals with at least one of the less common T alleles were significantly more likely to have a higher BMI than individuals carrying the CC genotype. Cholesterol level (without medication use) at baseline was significantly associated with the *PPARG* rs1175543, the *PPARG* rs709158, and the *PPARD* rs2016520 SNPs. Specifically, individuals carrying the *PPARG* rs1175543 AA, the *PPARG* rs709158 AA, or the *PPARD* rs2016520 CT or TT genotypes were significantly more likely to be categorized as having baseline cholesterol levels of 240 mg/dL or greater compared to those with the *PPARG* rs1175543 AG or GG, the *PPARG* rs709158 AG or GG, or the *PPARD* rs2016520 CC genotypes, respectively. Further, participants carrying the *PPARG* rs4684847 CT or TT genotypes were more likely to be categorized as being prehypertensive or hypertensive at baseline compared to participants carrying the CC genotype. No statistically significantly associations were observed between any of the *PPAR* sNPs and high blood pressure, high cholesterol, or diabetes diagnoses over the entire followup period. To note, after adjustment for multiple testing, none of the associations between the SNPs and the cardiovascular risk factors were statistically significant.

There were no statistically significant associations between the *PPAR* SNPs and cardiovascular deaths or events (including nonfatal events combined and MI alone) (see Table 3). A 40% reduction in the risk of premature cardiovascular death was observed for individuals with the *PPARG* rs4684847 CT or TT genotype compared to the CC genotype; however, the confidence interval was wide due to a small number of deaths (see Table 4). There were no statistically significant associations for premature death (all cause and cardiovascular-related) and *PPARG* rs709158, *PPARG* rs1175543, *PPARG* Pro12Ala, and *PPARD* rs2016520.

## 4. DISCUSSION

In general, the findings from this prospective, community-based cohort study indicate that the selected SNPs in *PPAR* genes are not associated with overall and premature cardiovascular morbidity and mortality. Further, with a few exceptions, we found that the selected *PPAR* SNPs were not associated with risk factors of cardiovascular disease. These findings were consistent among both men and women, among those with a diagnosis of diabetes, and in strata defined by baseline BMI.

Data on *PPAR* polymorphisms and cardiovascular disease and cardiovascular disease risk factors are limited and inconsistent. The most studied *PPAR* polymorphism, *PPARG* Pro12Ala, has been shown to be associated with reduced *PPARG* activity [14], and initial studies reported a lower risk of type 2 diabetes associated with the Ala12 allele [14]. This association was observed in some [15–19], but not all [20–23], subsequent studies. The lack of consistency also extends to studies examining the *PPARG* Pro12Ala polymorphism and cardiovascular disease events: one prospective cohort study examining the *PPARG* Pro12Ala polymorphism and cardiovascular disease suggested a decreased risk of CHD among carriers of the Ala12 allele [8], while others have shown no association [9,10,24] or an increase in risk [9, 25]. Although our study was limited by the small number of cardiovascular events, our results are consistent with those previous studies that have shown no association. This finding, as well as the data showing a lack of association between *PPARG* Pro12Ala and cardiovascular risk factors, suggests that this SNP is not involved in the development of cardiovascular disease in this population.

While most of the analyses conducted resulted in null findings, statistically significant age-adjusted associations were observed for *PPARG* rs4684847 with both baseline BMI and blood pressure, and for *PPARG* rs709158, *PPARG* rs1175543 and *PPARD* rs2016520 with baseline cholesterol levels. These associations were not statistically significant when self-reported blood pressure or high cholesterol at any time point over the study period were considered as outcome variables. Further, the statistical significance of the associations disappeared after correction for multiple testing. However, this may be due to a lack of statistical power. To our knowledge, these polymorphisms have not been examined in relation to cardiovascular disease or cardiovascular disease risk factors. Further, the functionalities of these polymorphisms are unknown. Although located in intron regions, these polymorphisms may affect enzyme distribution or other physiological functions related to cardiovascular health. Alternatively, it may be that the associations observed with these SNPs in this study are due to the polymorphisms being in linkage disequilibrium with other functional polymorphisms in the respective regions.

Several limitations of this study must be considered when interpreting the results. First, all of the followup data on cardiovascular risk factors and events were based on self-report. Because of this, there were some missing data for those who did not complete any of the followup questionnaires. Further, among those who did complete the questionnaires, there is the possibility of misclassification; this would likely be nondifferential, resulting in a dilution of the true risk estimate. However, in another investigation of this population, self-report of incidence of MI was compared to data gathered from on ongoing county-wide surveillance of MI events documented by hospital review and the sensitivity and specificity of reports were in excess of 94% (HY Huang, personal communication). A second limitation is that because of the racial homogeneity of the sample we were not able to explore potential racial differences in the associations between the *PPAR* polymorphisms and cardiovascular disease. In a previous publication [12], we did find, however, racial differences in the prevalence of the *PPAR* polymorphisms in this study that are consistent with those published in the SNP500 and dbSNP databases.

## 5. CONCLUSIONS

The results from this prospective, community-based cohort study showed no association between the selected *PPAR* polymorphisms and cardiovascular morbidity and mortality. In addition, in general, no statistically significant associations were observed between the *PPAR* polymorphisms and cardiovascular risk factors. Future studies should be conducted to confirm these findings and to explore the associations in populations with greater racial and ethnic heterogeneity.

## Figures and Tables

**Table 1:** Characteristics of study sample by gender, *N* = 9364, *P* value derived from *χ*2 test for categorical variables, Student’s t-test for continuous variables.

	Female *N* = 5776 (%)	Male *N* = 3588 (%)	Total (%)	*P* value
Age, mean(SD)	53.2 (15.4)	52.9 (15.5)	53.1 (15.4)	.3974
Education				.0029
<12	24.5	25.0	24.7	
12	47.3	44.0	46.0	
>12	28.2	31.0	29.3	
Missing, *n*	4	1	5	

BMI, kg/m^2^, mean (SD)	26.1 (5.3)	26.6 (4.0)	26.3 (4.9)	<.0001
BMI, kg/m^2^				<.0001
< 25	48.8	34.0	43.1	
25–30	30.8	48.8	37.7	
>30	20.4	17.3	19.2	
Missing, *n*	11	1	12	

Smoking status				<.0001
Never	62.5	40.3	54.0	
Former	21.4	42.7	29.6	
Current	16.1	17.0	16.4	
Missing, *n*			0	

Cholesterol no Rx, mg/dL				<.0001
≤200	41.3	48.2	43.9	
200–239	37.1	36.9	37.0	
≥240	21.6	14.9	19.1	

Cholesterol with Rx, mg/dL				<.0001
≤200	15.7	36.0	23.1	
200–239	43.9	36.5	41.2	
≥240	40.4	27.5	35.7	

Blood pressure				<.0001
Normal	30.2	15.6	24.6	
Prehypertensive	56.4	67.6	60.7	
Hypertensive	13.4	16.8	14.7	
Missing, *n*	5	6	11	

*PPARG* rs4684847				.0910
CC	78.8	77.3	78.2	
CT/TT	21.2	22.7	21.8	
Missing, *n*	188	120	308	

*PPARG* rs709158				.4741
AA	39.6	40.3	39.8	
AG/GG	60.4	59.7	60.2	
Missing, *n*	134	98	232	

*PPARG* rs1175543				.9218
AA	40.2	40.1	40.2	
AG/GG	59.8	59.9	59.8	
Missing, *n*	136	91	227	

*PPARD* rs2016520				.5240
CC	64.6	64.0	64.4	
CT/TT	35.4	36.0	35.6	
Missing, *n*	146	104	250	

*PPARG* rs1801282				.2728
Pro/Pro	78.4	77.5	78.1	
Pro/Ala or Ala/Ala	21.6	22.5	21.9	
Missing, *n*	156	106	262	

**Table 2(a):** 

Risk factors	*PPARG* rs4684847			*PPARG* rs709158			*PPARG* 1175543		
	CC	CT/ TT	*P* value	AA	AG/GG	*P* value	AA	AG/GG	*P* value

*Baseline characteristics*									
Age, mean(SD)	53.0 (15.5)	53.4 (15.4)	.3571	52.7 (15.4)	53.3 (15.4)	.0780	52.9 (15.5)	53.3 (15.3)	.1910
BMI, kg/m^2^, *n* (%)			.0203			.8458			.9777
								
<25	3097 (79)	815 (21)		1562 (40)	2372 (60)		1575 (40)	2366 (60)	
25–30	2645 (78)	745 (22)		1392 (40)	2048 (60)		1393 (41)	2037 (59)	
>30	1331 (76)	412 (24)		681 (39)	1067 (61)		696 (40)	1059 (60)	

Cholesterol no Rx, mg/dL,			.7144			.0370			.0198
*n*(%)									
≤200	2956 (79)	791 (21)		1469 (39)	2295 (61)		1481 (39)	2284 (61)	
200–239	2443 (78)	702 (22)		1274 (40)	1897 (60)		1281 (40)	1904 (60)	
≥240	1275 (79)	346 (21)		674 (41)	964 (59)		688 (42)	943 (58)	

Cholesterol with Rx, mg/dL,			.8997			.4595			.3418
*n* (%)									
≤200	82 (76)	26 (24)		40 (36)	71 (64)		37 (34)	71 (66)	
200–239	141 (74)	50 (26)		78 (39)	121 (61)		79 (40)	119 (60)	
≥240	125 (75)	42 (25)		70 (41)	102 (59)		70 (41)	102 (59)	

Blood pressure, *n* (%)			.0248			.0513			.0509
Normal	1777 (79)	460 (21)		875 (39)	1369 (61)		882 (39)	1362 (61)	
Prehypertensive	4290 (78)	1193 (22)		2209 (40)	3338 (60)		2221 (40)	3322 (60)	
Hypertensive	1005 (76)	320 (24)		549 (41)	782 (59)		561 (42)	779 (58)	

*Combined outcomes*									
Diabetes, *n* (%)			.7845			.8617			.5499
No	4504 (78)	1243 (22)		2325 (40)	3466 (60)		2350 (41)	3451 (59)	
Yes	821 (78)	225 (22)		420 (40)	643 (60)		418 (39)	648 (61)	

High cholesterol, *n* (%)			.3452			.4527			.5670
No	2502 (78)	706 (22)		1280 (40)	1944 (60)		1296 (40)	1938 (60)	
Yes	2712 (79)	730 (21)		1408 (40)	2081 (60)		1414 (41)	2075 (59)	

High blood pressure, *n* (%)			.6258			.2150			.2724
No	2441 (79)	660 (21)		1287 (41)	1834 (59)		1293 (41)	1829 (59)	
Yes	3379 (78)	969 (22)		1720 (39)	2675 (61)		1741 (40)	2663 (60)	

**Table 2(b):** 

Risk factors	*PPARD* rs2016520			*PPARG* rs1801282		
	CC	CT/TT	*P* value	Pro/Pro	Pro/Ala or Ala/Ala	*P* value

Age, mean(SD)	53.0 (15.4)	53.0 (15.5)	.9640	52.9 (15.4)	53.2 (15.3)	.4587
BMI, kg/m^2^, *n* (%)			.9138			.1280
<25	2534 (64)	1389 (36)		3085 (79)	832 (21)	
25–30	2190 (64)	1246 (36)		2668 (78)	757 (22)	
>30	1136 (65)	609 (35)		1346 (77)	403 (23)	

Cholesterol no Rx, mg/dL, *n* (%)			.0161			.7401
≤200	2475 (66)	1290 (34)		2953 (78)	811 (22)	
200–239	2011 (63)	1161 (37)		2467 (78)	705 (22)	
≥240	1019 (63)	604 (37)		1261 (78)	354 (22)	

Cholesterol with Rx, mg/dL, *n* (%)			.3821			.6233
≤200	74 (67)	37 (33)		86 (80)	22 (20)	
200–239	135 (69)	62 (31)		148 (76)	48 (24)	
≥240	106 (63)	63 (37)		128 (76)	40 (24)	

Blood pressure, *n* (%)			.8076			.2636
Normal	1441 (64)	812 (36)		1769 (79)	483 (21)	
Prehypertensive	3578 (65)	1939 (35)		4312 (78)	1199 (22)	
Hypertensive	842 (63)	491 (37)		1016 (77)	312 (23)	

*Combined outcomes*						
Diabetes, *n* (%)			.6185			.1278
No	3747 (65)	2058 (35)		4517 (78)	1275 (22)	
Yes	675 (64)	383 (36)		841 (80)	212 (20)	

High cholesterol, *n* (%)			.3178			.3691
No	2060 (64)	1175 (36)		2516 (78)	713 (22)	
Yes	2263 (65)	1225 (35)		2732 (79)	740 (21)	

High blood pressure, *n* (%)			.9412			.8331
No	2006 (64)	1121 (36)		2462 (78)	676 (22)	
Yes	2821 (64)	1564 (36)		3384 (78)	971 (22)	

**Table 3:** Age-adjusted hazard ratios (HR) and 95% confidence intervals (95% CI) of all-cause and cardiovascular mortality and nonfatal cardiovascular events for 5 PPAR polymorphisms. Person years: cumulative person years of follow up. RR (95% CI): relative risk using poisson regression.

		All-cause death		CV-related death		Nonfatal CV events		Fatal and nonfatal CV events
SNPs	Person years	*n*	HR (95% CI)	*n*	HR (95% CI)	*n*	RR (95% CI)	RR (95% CI)

*PPARG* rs4684847								
CC	92827	1705	1.00 (reference)	416	1.00 (reference)	2351	1.00 (reference)	1.00 (reference)
CT/TT	25880	474	0.95 (0.85–1.05)	114	0.90 (0.73–1.11)	679	1.00 (0.73–1.37)	0.88 (0.67–1.18)

*PPARG* rs709158								
AA	47812	850	1.00 (reference)	205	1.00 (reference)	1202	1.00 (reference)	1.00 (reference)
AG/GG	71972	1328	0.99 (0.91–1.09)	324	1.02 (0.86–1.20)	1843	0.98 (0.75–1.28)	0.98 (0.77–1.24)

*PPARG* rs1175543								
AA	48172	867	1.00 (reference)	213	1.00 (reference)	1183	1.00 (reference)	1.00 (reference)
AG/GG	71627	1331	1.0 (0.92–1.09)	316	0.99 (0.83–1.18)	1864	1.03 (0.79–1.34)	1.00 (0.79–1.28)

*PPARD* rs2016520								
CC	77042	1386	1.00 (reference)	342	1.00 (reference)	2027	1.00 (reference)	1.00 (reference)
CT/TT	42593	766	1.03 (0.94–1.12)	178	1.02 (0.85–1.23)	1003	0.91 (0.69–1.19)	0.98 (0.76–1.26)

*PPARG* rs1801282								
Pro/Pro	93254	1696	1.00 (reference)	408	1.00 (reference)	2350	1.00 (reference)	1.00 (reference)
Pro/Ala or Ala/Ala	26196	456	0.96 (0.86–1.06)	112	0.97 (0.79–1.20)	656	0.98 (0.71–1.35)	0.96 (0.72–1.28)

**Table 4:** Age-adjusted hazard ratios (HR) and 95% confidence intervals (95% CI) of premature (age < 65 years) all-cause and cardiovascular mortality for 5 PPAR SNPs. Person years: cumulative person years of follow up.

		All-cause death		CV-related death	
SNPs	Person years	*n*	HR (95% CI)	*n*	HR (95% CI)

*PPARG* rs4684847					
CC	43445	231	1.00 (reference)	27	1.00 (reference)
CT/TT	11743	58	1.05 (0.78–1.40)	4	0.60 (0.21–1.72)

*PPARG* rs709158					
AA	23078	115	1.00 (reference)	13	1.00 (reference)
AG/GG	32822	175	1.05 (0.83–1.33)	18	0.95 (0.47–1.95)

*PPARG* rs1175543					
AA	22875	115	1.00 (reference)	14	1.00 (reference)
AG/GG	32859	175	1.04 (0.82–1.32)	17	0.83 (0.41–1.68)

*PPARD* rs2016520					
CC	36389	186	1.00 (reference)	21	1.00 (reference)
CT/TT	19940	99	0.98 (0.77–1.25)	9	0.79 (0.36–1.71)

*PPARG* rs1801282					
Pro/Pro	44263	236	1.00 (reference)	25	1.00 (reference)
Pro/Ala or Ala/Ala	12247	50	0.90 (0.66–1.23)	5	0.79 (0.30–2.09)
